# Bringing home the benefits: do pro-family employee benefits mitigate the risk of depression from competing workplace and domestic labor roles?

**DOI:** 10.1093/aje/kwae055

**Published:** 2024-04-26

**Authors:** Jonathan M Platt, Lisa Bates, Justin Jager, Katie A McLaughlin, Katherine M Keyes

**Affiliations:** Department of Epidemiology, College of Public Health, The University of Iowa, Iowa City, IA 52242, United States; Department of Epidemiology, Mailman School of Public Health, Columbia University, New York, NY 10032, United States; T. Denny School of Social and Family Dynamics, Arizona State University, Tempe, AZ 85287, United States; Department of Psychology, Harvard University, Cambridge, MA 02138, United States; Department of Epidemiology, Mailman School of Public Health, Columbia University, New York, NY 10032, United States

**Keywords:** depression, sex/gender, labor, employee benefits, competing roles

## Abstract

Despite significant historical progress toward sex/gender parity in employment status in the United States, women remain more likely to provide domestic labor, creating role competition which may increase depression symptoms. Pro-family employee benefits may minimize the stress of competing roles. We tested whether depressive symptoms were higher among women with competing roles versus without competing roles and whether this effect was greater among women without (vs with) pro-family benefits. Data included employed women (*n* = 9884 person-years) surveyed across 4 waves (2010, 2015, 2017, and 2019) of the National Longitudinal Survey 1997. Depression symptoms were measured with the 5-item short version of the Mental Health Inventory (MHI-5). The effect of interaction between competing roles and pro-family employee benefits on depressive symptoms was also compared with that of non–family-related benefits, using marginal structural models to estimate longitudinal effects in the presence of time-varying confounding. MHI-5 scores were 0.56 points higher (95% CI, 0.15-0.97) among women in competing roles (vs not). Among women without pro-family benefits, competing roles increased MHI-5 scores by 6.10 points (95% CI, 1.14-11.1). In contrast, there was no association between competing roles and MHI-5 scores among women with access to these benefits (MHI-5 difference = 0.44; 95% CI, −0.2 to 1.0). Results were similar for non–family-related benefits. Dual workplace and domestic labor role competition increases women’s depression symptoms, though broad availability of workplace benefits may attenuate that risk.

This article is part of a Special Collection on Mental Health.

## Introduction

Throughout the 20th century, women’s participation in the labor force increased in the United States.^1^ Today, women are as likely as men to be employed full-time.^2^ Domestic roles have changed as well.^3^^‑^^5^ However, in spite of these historic changes, significant sex/gender disparities in workplace and domestic labor remain.^6^ Among men and women with similar workplace obligations, women still spend more time on daily domestic labor and childcare,^7^^‑^^9^ even among women who out-earn their male spouses.^10^

As a result of these incomplete changes, women are now more likely to hold competing workplace and domestic roles, which may influence their mental health, including risk of depression.^11^ Depression in the United States is common, impairing, and approximately twice as likely to occur among women as among men.^12^^,^^13^ However, there is limited consensus on the impact of concurrent role obligations on depression. Some studies have found that multiple roles reduce depressive symptoms by increasing sources of social and material resources.^14^^‑^^18^ Others have found that the potential for conflict- and overload-related stress increases with the number of roles, thus increasing depression symptoms.^19^^‑^^22^ More specifically, holding multiple roles may negatively influence mental health when the time, energy, and attention resources needed to satisfy one role compete with the resources needed to satisfy another role.^23^

The 2020 COVID-19 pandemic led to a widespread and profound convergence of work and domestic lives. To mitigate exposure to and spread of infection, many employers rapidly shifted to remote work settings, instructing their employees to work from home. Schools closed, and students attended school remotely from home. In many families, all family members spent significantly more time in the home, likely increasing the risk of competing roles among women in particular, who are more likely to fulfill childcare needs during the workday.^24^^,^^25^

Employers have increasingly offered pro-family employee benefits, such as paid family leave and flexible working schedules, as a way to address the burden from competing roles and to retain employees with children.^26^ These are distinct from other employee benefits that are not explicitly intended to buffer the effects of competing roles (eg, retirement pensions, health insurance),^27^ although employee benefits cluster together by occupation and industry.^28^ Evidence suggests that pro-family benefits may mitigate the burden of competing role demands among female employees. Women with access to pro-family benefits are less likely to experience work–family conflict,^29^ competing responsibilities,^30^ and job dissatisfaction^31^ and as a result are more likely to remain employed^32^^,^^33^ and to maintain prechildbirth work hours.^34^

Pro-family benefits are associated with better physical health^35^ and general well-being.^36^ Positive mental health effects have also been shown^37^; however, evidence is limited in at least 3 ways. First, studies typically consider exposure to a single policy, such as the availability/length of paid family leave^38^^,^^39^ or work schedule flexibility,^40^ rather than exposure to multiple policies in the same population with attention to the potential cumulative effects of multiple benefits. Second, studies have not tested the specificity of the effect of pro-family benefits by comparing them with the effects of policies that are not family-related. Workplace benefits, as a whole, may improve employee mental health through alternative pathways,^41^^‑^^43^ so in effect, the broader workplace context may confound any observed association between pro-family benefits and depression. Comparing the effects of pro-family benefits with those of other benefits will interrogate this source of confounding and strengthen the theoretical claim that pro-family policies mitigate the negative effects of competing roles to reduce depression risk. Third, studies often rely on cross-sectional data.^44^^‑^^46^ Longitudinal studies are necessary to understand the potential bias from reverse causation, selection, and time-varying confounding. If depression and exposure to competing roles both increased the risk of selection out of the workforce, effect estimates of competing roles would be spuriously attenuated. Selection bias might also arise if individuals with depression and competing roles were less likely to work in jobs with employee benefits, attenuating any mitigating effects of benefits. Selection bias may also arise due to inequitable access to jobs in which benefits are offered to employees. In addition, important confounding variables related to employment (eg, income, hours of work) and the domestic context (eg, number of children, marital status) may change over time. Accounting for these changes strengthens the internal validity of model estimates.

To address these limitations, in the present study we aimed to: (1) estimate the relationship between competing roles (working and raising children vs working and *not* raising children) and depression; (2) assess whether that relationship varied by the presence of any pro-family employee benefits, as well as the number of available benefits; (3) assess whether any observed buffering effect by pro-family benefits was similar to the buffering effects due to non–family-related benefits; and (4) estimate the potential impact of 2 important sources of selection bias: selection out of employment and selection out of jobs with available employee benefits.

## Methods

### Sample

Data were from the National Longitudinal Survey 1997 cohort, one in a series of prospective studies of the employment, education, domestic, and health experiences of American adults.^47^ Detailed information for each study has been previously published.^48^^‑^^52^ The analytical sample comprised data from the 7 most recent interviews that included outcome data (the 2010, 2015, 2017, and 2019 waves). The sample ranged in age from 26-30 years in 2010 to 35-39 years in 2019, representing the ages at which childbearing and employment are both highly salient and likely to overlap as competing roles. The sample included only female respondents who were employed at each wave, to ensure that the entire sample had the potential to be exposed to employee benefits and to avoid structural positivity violations. Respondents could “re-enter” the sample after a period of unemployment. Those who were unemployed, self-employed in an unincorporated business, or enlisted in the military were not asked about employee benefits. The study sample included 2374 women in 2010 and 2499 in 2019. In total, the analytical data set comprised 9884 person-years. Figure S1 presents a study flow chart with sample sizes and reasons for exclusion at each wave.

### Measures

#### Mental Health Inventory depression scale

The primary outcome was a 5-item short version of the Mental Health Inventory (MHI-5),^53^ which measures symptoms of depression and anxiety (nervousness, depressed affect) and positive aspects of mental health (feeling calm, feeling happy) in the past 4 weeks (score range, 0-15; higher scores indicate more symptomatology).^54^ The MHI-5 has reasonable validity, is an effective screening instrument for mood disorders in the general population,^55^^,^^56^ and has been validated against clinical interviews as a measure of depression.^57^^,^^58^

#### Competing roles

The independent variable was “competing roles,” defined as being currently employed full-time and self-identifying as the cohabiting parent of at least 1 child aged <18 years. Full-time employment was defined by the National Longitudinal Survey administrators as working at least 35 hours per week. The comparison group for persons with competing roles was defined as those who were currently employed with no children under age 18.

#### Employee benefits

Respondents reported the availability (yes/no) of 9 employee benefits in their current job at each interview. Benefits were classified as either pro-family or non–family-related, concordant with previous research, based on their hypothesized potential to alleviate the burden and stress due to competing workplace and domestic roles.^59^ Pro-family benefits included family leave, flexible work scheduling, and employer-provided/subsidized childcare. Non–family-related benefits included dental insurance, medical insurance, life insurance, profit-sharing, retirement pension programs, and training/educational opportunities. Each group of benefits was considered as a binary variable (eg, any benefits vs no benefits), as a count variable (0 vs 1 or ≥2), and individually in a sensitivity analysis (described below).

### Confounding and selection bias

**Figure 1:**
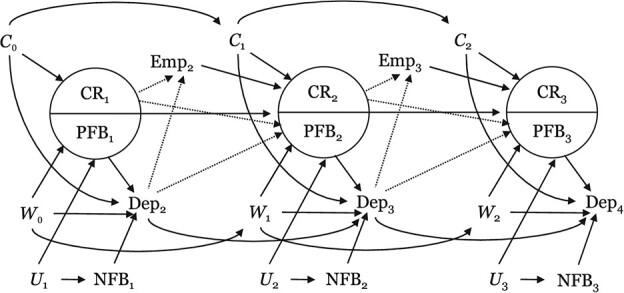
Hypothesized structure of confounding (*C*) and selection bias in a study of pro-family employee benefits (PFB) and risk of depression (Dep; assessed via MHI-5 scores) from competing workplace and domestic labor roles, National Longitudinal Survey 1997, 2010-2019. Solid arrows represent sources of potential confounding; dotted arrows represent sources of potential selection bias. Possible confounders include age, education, income, weekly work hours, marital status, and race/ethnicity. Non–family-related benefits (NFB) are intermediate variables between unmeasured confounders and PFB. CR, competing roles; Emp, employment status; MHI-5, Mental Health Inventory, 5-item short version; *U*, unmeasured variable representing an alternative pathway between PFB and MHI-5 scores; *W*, potential confounders of the relationship between PFB and Dep (age, race/ethnicity, weekly work hours, number of children, number of children aged <5 years, income, education, employer type, and industry).

Figure 1 presents the analytical model, including the structure of confounding and selection bias over time. Corresponding variable labels in the directed acyclic graph are noted in parentheses. Potential confounders (*C*) of the relationship between competing roles (CR) and MHI-5 scores (Dep) included time-invariant age (continuous; years) and race/ethnicity (Hispanic or Latino, Black, non-Black/non-Hispanic, or mixed-race/non-Hispanic) and time-varying years of education, weekly number of hours of paid work, annual income (continuous; dollars), and marital status (never married, currently married, or formerly married). Race/ethnicity was recorded only at study baseline and thus could not be operationalized as time-varying. Potential confounders (*W*) of the relationship between pro-family employee benefits (PFB) and MHI-5 scores (Dep) included time-invariant age and race/ethnicity and time-varying education, number of children (1, 2, or ≥3), number of children under age 5 years (0, 1, or ≥2), weekly hours of paid work, employer type (government, private sector, or nonprofit), income, and industry (21 categories, listed in Table S1). Additionally, pro-family benefits may be related to MHI-5 scores through unmeasured pathways not related to competing risk, such as measures of perceived job quality, stability, or prestige. We attempted to control this unmeasured bias by adjusting for non–family-related benefits (NFB) as intermediate variables between unmeasured confounders and pro-family benefits.

Only employed respondents reported on the availability of employee benefits, which may have introduced selection bias based on employment status. In a sensitivity analysis, we attempted to quantify the magnitude of this source of selection bias by estimating the strength of the association between selection out of employment related to MHI-5 symptom scores (Dep-Emp) and competing role status (CR-Emp) in prior waves. Additionally, to account for potential bias from differential selection into jobs with pro-family benefits, we examined the probability of holding a job with pro-family benefits based on prior MHI-5 symptom scores (Dep-PFB) and competing role status (CR-PFB). Selection pathways are denoted with dotted lines in the directed acyclic graph.

### Multiple imputation

Within the sample, 15% of MHI-5 scores were missing. To minimize this loss of data, we imputed missing data using multivariate imputation by chained equations. Imputation models included all confounding variables and observed MHI-5 scores. Ten data sets were imputed and combined in the analytical models with corrected SEs.^60^ Imputed model estimates were compared with nonimputed estimates in order to examine the robustness of analytical models to the degree of missing data.

### Analysis

The distributions of the study variables were summarized as mean values and SDs for continuous variables and as proportions for categorical variables for all available person-time over the study period. Analysis of variance and χ^2^ statistics were used to test whether the means or proportions of each variable differed by competing role status.

The analytical aims were implemented using a series of marginal structural models, in order to appropriately control for confounding over time while avoiding overadjustment due to controlling on variables that may act as confounders and mediators, depending on the exposure time point.^61^ The observed data were reweighted at each wave by the inverse probability of treatment (ie, having competing roles), conditional on the confounding variables at that wave.^62^ Weights were stabilized to attenuate the influence of outliers with large weights. The final models were weighted by the product of weights at each wave. Linear models were used to estimate continuous MHI-5 score differences with cluster-robust SEs to account for uneven follow-up periods and the clustering of observations within individuals over time.^63^

The first aim was to estimate the relationship between competing roles and depression and the relationship between any pro-family benefits and depression in both unadjusted and adjusted models. The second aim was to assess whether the relationship between competing roles and depression varied by the presence of any pro-family benefits as well as the number of available benefits. This was accomplished with 2 steps. First, a model tested for interaction between competing roles and pro-family benefits, to examine whether pro-family employee benefits buffered the effect of competing roles on risk of depression symptoms. Interaction was tested using cross-product methods in linear models. Second, in addition to directly testing for interaction, models were stratified to examine the magnitude of the risk from competing roles in the presence versus absence (and count) of pro-family benefits, with adjustment for non–family-related benefits.

Third, to assess whether any observed buffering effect by pro-family benefits was similar to the buffering effects due to non–family-related benefits, we estimated the depression risk of competing roles, stratified by exposure to non–family-related employee benefits (both binary and counts), with adjustment for pro-family benefits.

To further probe the effects of employee benefits, we estimated the multiplicative interaction coefficient and 99% CI for each individual benefit in a sensitivity analysis. Each benefit was adjusted for all other benefits, to conservatively estimate the magnitude of each benefit underlying the analysis of grouped benefits.

Finally, to examine the potential bias due to selection out of employment and selection into jobs with available employee benefits, based on prior depressive symptoms and competing role status, we estimated the risk of being unemployed and reporting available pro-family benefits, based on prior MHI-5 symptom scores, and competing role status.

## Results

The distributions of all study variables according to competing role status and availability of pro-family employee benefits over the study period are presented in Table 1. Overall, the prevalence of competing roles was 69%, and the prevalence of any pro-family benefits was 87%. Compared with those not in competing roles, women in competing roles were older, more likely to be married, and more likely to identify as non-Black/non-Hispanic. Education and employment differences were also apparent. Women in competing roles were more likely to have a college degree or more, to have a higher annual income ($26 900 vs $42 200), and to work more hours per week. Further, these socioeconomic characteristics were lowest among women in jobs without pro-family benefits. MHI-5 scores were highest among those with no competing roles with jobs lacking pro-family benefits. The availability of non–family-related benefits was highly correlated with pro-family benefits.

**Table 1:** Distributions of study variables for all available person-time (*n* = 9884), overall and by competing role status and availability of pro-family employee benefits, National Longitudinal Survey 1997, 2010-2019

			**Competing role status**			
**Characteristic**	**Overall**		**Competing roles**		**No competing roles**	
	**No PFB** **(*n* = 1297)**	**Any PFB** **(*n* = 8587)**	**No PFB** **(*n* = 909)**	**Any PFB** **(*n* = 5959)**	**No PFB** **(*n* = 388)**	**Any PFB** **(*n* = 2628)**
Mean (SD) age, y	31.5 (3.60)	33.5 (3.51)	31.7 (3.55)	33.9 (3.28)	31.0 (3.68)	32.5 (3.79)
Mean (SD) no. of children aged <18 y	2.26 (1.21)	2.12 (1.09)	2.26 (1.21)	2.12 (1.09)	0 (0)	0 (0)
Mean (SD) annual income, per $1000	26.9 (13)	42.2 (32.3)	25.9 (11.9)	40.6 (31.9)	29.3 (15.2)	46 (32.8)
Mean (SD) weekly hours of paid of work	38.0 (5.94)	40.4 (6.73)	37.9 (6.03)	40.0 (6.56)	38.1 (5.73)	41.2 (7.04)
Mean (SD) no. of childcare sources			1.54 (0.803)	1.85 (1.01)	0 (0)	0 (0)
Mean (SD) MHI-5 score	4.58 (2.67)	3.80 (2.36)	4.50 (2.65)	3.67 (2.34)	4.75 (2.72)	4.11 (2.37)
Highest attained education, no. (%)						
Less than a HS diploma	356 (27.4)	1155 (13.5)	283 (31.1)	926 (15.5)	73 (18.8)	229 (8.7)
HS diploma	522 (40.2)	2859 (33.3)	381 (41.9)	2221 (37.3)	141 (36.3)	638 (24.3)
College degree	324 (25.0)	3055 (35.6)	200 (22.0)	1941 (32.6)	124 (32.0)	1114 (42.4)
Graduate degree	89 (6.9)	1486 (17.3)	41 (4.5)	844 (14.2)	48 (12.4)	642 (24.4)
Marital status, no. (%)						
Never married	695 (53.6)	3555 (41.4)	438 (48.2)	1991 (33.4)	257 (66.2)	1564 (59.5)
Married	428 (33.0)	3901 (45.4)	346 (38.1)	3108 (52.2)	82 (21.1)	793 (30.2)
Separated	31 (2.4)	163 (1.9)	21 (2.3)	134 (2.2)	10 (2.6)	29 (1.1)
Divorced	133 (10.3)	938 (10.9)	97 (10.7)	704 (11.8)	36 (9.3)	234 (8.9)
Widowed	10 (0.8)	30 (0.3)	7 (0.8)	22 (0.4)	3 (0.8)	8 (0.3)
Race/ethnicity, no. (%)						
Black	411 (31.7)	2448 (28.5)	309 (34.0)	1847 (31.0)	102 (26.3)	601 (22.9)
Hispanic	302 (23.3)	1763 (20.5)	220 (24.2)	1312 (22.0)	82 (21.1)	451 (17.2)
Mixed-race (non-Hispanic)	10 (0.8)	68 (0.8)	8 (0.9)	43 (0.7)	2 (0.5)	25 (1.0)
Non-Black/non-Hispanic	574 (44.3)	4308 (50.2)	372 (40.9)	2757 (46.3)	202 (52.1)	1551 (59.0)
Any non–family-related benefits, no. (%)	288 (22.2)	7736 (90.1)	207 (22.8)	5363 (90.0)	81 (20.9)	2373 (90.3)

Abbreviations: HS, high school; MHI-5, Mental Health Inventory, 5-item short version; PFB, pro-family employee benefits.

The effects of competing roles and pro-family benefits on MHI-5 scores (aim 1) are presented in Table 2. After adjustment, women in competing roles had a 0.56-point higher MHI-5 score (95% CI, 0.15-0.97) than women not in competing roles. Women with any available pro-family benefits had a 0.83-point lower MHI-5 score (95% CI, −1.36 to −0.31) than women without pro-family benefits.

**Table 2:** Difference in MHI-5 symptom score among women spending time in competing roles and women with any pro-family employee benefits, National Longitudinal Survey 1997, 2010-2019

**Variable**	**MHI-5 score difference (95% CI)**	
	**Unadjusted**	**Adjusted**
Competing roles^a^	0.72 (0.32 to 1.12)	0.56 (0.15 to 0.97)^b^
Any pro-family employee benefits^c^	−0.70 (−1.07 to −0.33)	−0.83 (−1.36 to −0.31)^d^

Abbreviation: MHI-5, Mental Health Inventory, 5-item short version.

^a^Competing roles were defined as working for pay with children living in the respondent’s household (referent: working with no children living in the household).

^b^ Adjusted for age, race/ethnicity, years of education, number of weekly hours of paid work, income, and marital status.

^c^ Referent: no pro-family benefits.

^d^ Adjusted for age, race/ethnicity, years of education, number of children, number of children under age 5 years, weekly hours of paid work, employer type, income, and industry.

**Figure 2:**
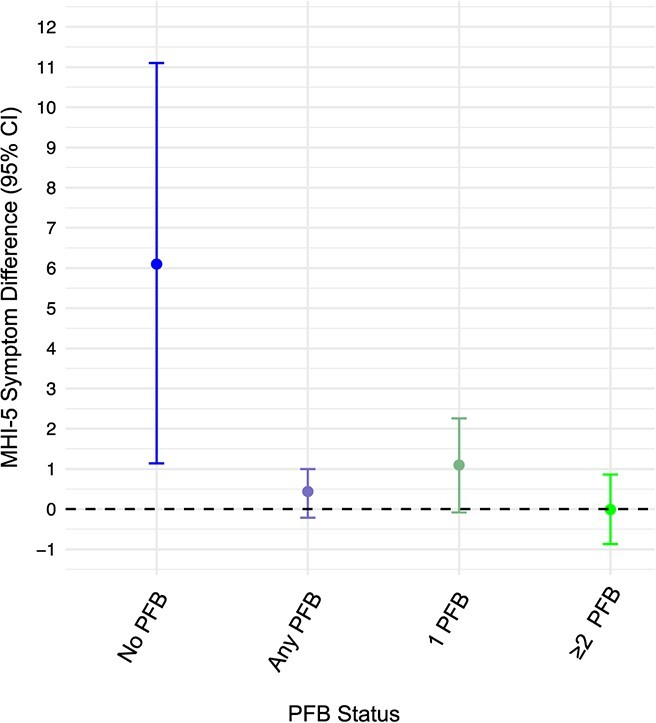
MHI-5 symptom score differences among women in competing roles versus those not in competing roles, according to the availability (any vs none) and number of pro-family employee benefits (PFB), National Longitudinal Survey 1997, 2010-2019. Results were adjusted for age, race/ethnicity, hours of paid work per week, employer type, industry, education, number of children, number of children aged <5 years, and non–family-related benefits (NFB). Competing roles were defined as working for pay with children living in the respondent’s household (referent: working with no children living in the household). Interaction test (H_0_: competing roles × PFB = 0): interaction estimate = −0.51 (*P* =.017) without adjustment for NFB and estimate = −0.44 (*P* =.023) with adjustment for NFB. Bars show 95% CIs. CES-D, Center for Epidemiologic Studies Depression Scale; MHI, Mental Health Inventory, 5-item short version.

Figure 2 (and Table S2) presents interaction tests and stratified analysis of the effects of competing roles on MHI-5 scores in the absence (vs presence) of any pro-family benefits and the number of available benefits (aim 2). In the linear models, the interaction between pro-family benefits and competing roles was associated with MHI-5 scores, both unadjusted (β = −0.51, *P* =.017) and adjusted (β = −0.44, *P* =.023). In the absence of any available pro-family benefits, women in competing roles had 6.10-point higher MHI-5 scores (95% CI, 1.14-11.1) than those not in competing roles. In contrast, among women with access to any pro-family benefits, the association was largely attenuated, with a 95% CI that included the null (MHI-5 difference = 0.44; 95% CI, −0.2 to 1.0). Similar patterns were found among those reporting exactly 1 pro-family benefit (MHI-5 score difference = 1.1; 95% CI, −0.08 to 2.26) or 2 or more pro-family benefits (MHI-5 difference = −0.01; 95% CI, −0.87 to 0.86). The 95% CIs were appreciably wider among those without (vs with) pro-family benefits because of the low prevalence of no pro-family benefits.

**Figure 3:**
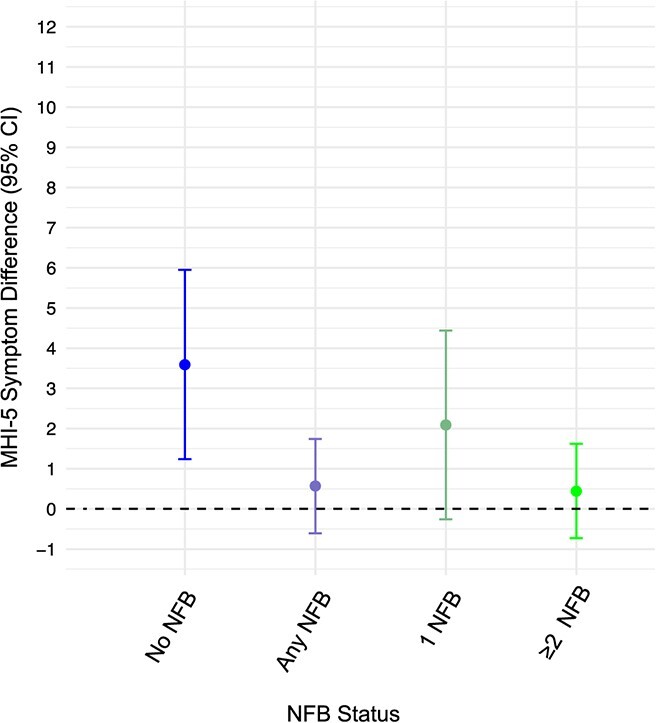
MHI-5 symptom score differences among women in competing roles versus those not in competing roles, according to the availability (any vs none) and number of non–family-related employee benefits (NFB), National Longitudinal Survey 1997, 2010-2019. Results were adjusted for age, race/ethnicity, hours of paid work per week, employer type, industry, education, number of children, number of children aged <5 years, and pro-family employee benefits. Competing roles were defined as working for pay with children living in the respondent’s household (referent: working with no children living in the household). Bars show 95% CIs. CES-D, Center for Epidemiologic Studies Depression Scale; MHI, Mental Health Inventory, 5-item short version.

Figure 3 (and Table S3) presents the effects of competing roles on MHI-5 scores stratified by the absence (vs presence) of any non–family-related benefits and the number of available benefits (aim 3). Women in competing roles without any non–family-related benefits had 3.59-point higher MHI-5 scores (95% CI, 1.24-5.95) than those not in competing roles. Among women with access to these benefits, the association between competing roles and MHI-5 symptoms was attenuated, with a 95% CI that included 0 (MHI-5 difference = 0.57; 95% CI, −0.61 to 1.74). Similar associations were found among women with exactly 1 non–family-related benefit (MHI-5 difference = 2.09; 95% CI, −0.26 to 4.44) and 2 or more benefits (MHI-5 difference = 0.44; 95% CI, −0.73 to 1.62). The 95% CIs were appreciably wider among women without (vs with) non–family-related benefits because of the low prevalence of no non–family-related benefits.

Compared with the data set with no imputation, multiply imputed model estimates were not meaningfully different, although SEs were slightly smaller overall.

The sensitivity analysis estimating the interaction between competing roles and individual employee benefits is presented in Table S4. Among pro-family benefits, the interaction coefficients for family leave and flexible work scheduling were −0.19 (99% CI, −0.37 to −0.01) and −0.18 (99% CI, −0.34 to −0.02), respectively. Among non–family-related benefits, the largest coefficients were for medical insurance (−0.41; 99% CI, −0.6 to −0.22) and dental insurance (−0.32; 99% CI, −0.5 to −0.15).

Table 3 shows the estimated magnitude of bias due to selection out of the workforce at each interview, based on the MHI-5 score and competing role status in the prior interview (aim 4). The risk of becoming unemployed among women exposed to competing roles in the prior interview ranged from 1.05 (95% CI, 1.03-1.07) in 2015 and 2019 to 0.99 (95% CI, 0.98-1.01) in 2010. The risk of becoming unemployed based on prior MHI-5 scores was effectively null at every wave. Selection into jobs with (vs without) pro-family benefits was also considered. The risk of reporting available pro-family benefits among those exposed to competing roles in the prior interview ranged from 1.03 (95% CI, 0.98-1.07) in 2019 to 0.96 (95% CI, 0.93-0.99) in 2010. The risk of reporting available pro-family benefits based on prior MHI-5 scores was effectively null at every year. Overall, selection risks were small due to competing roles and prior MHI-5 scores, suggesting that the results were not substantially influenced by selection bias.

**Table 3:** Relative risk (95% CI) of being unemployed and of reporting any available pro-family employee benefits, by competing role status and prior MHI-5 symptom score, National Longitudinal Survey 1997, 2010-2019

**Year**	**Unemployed** ^a^	**Any pro-family benefits** ^b^
*Competing Roles*		
2010	0.99 (0.98-1.01)	0.96 (0.93-0.99)
2015	1.05 (1.03-1.07)	1.00 (0.96-1.04)
2017	1.02 (1.01-1.03)	1.00 (0.96-1.03)
2019	1.05 (1.03-1.07)	1.03 (0.98-1.07)
*MHI-5 Score*		
2010	1.01 (1.01-1.01)	1.00 (0.99-1.00)
2015	1.00 (0.99-1.00)	1.00 (0.99-1.00)
2017	1.00 (0.99-1.00)	1.00 (0.99-1.00)
2019	1.00 (0.99-1.00)	1.00 (0.99-1.00)

Abbreviation: MHI-5, Mental Health Inventory, 5-item short version.

^a^ Referent: employed.

^b^ Referent: no pro-family benefits.

## Discussion

The present study examined the effect of competing sex/gender roles on women’s depression and whether that association was buffered by the availability of pro-family employee benefits. There were 4 central findings: (1) Among women in the workplace, those with competing gender roles reported higher levels of depression symptoms than those without competing roles; (2) the depression risk from competing roles was attenuated in the presence of pro-family benefits, supporting the buffering effects hypothesis; however, (3) the depression risk from competing roles was also attenuated in the presence of non–family-related employee benefits, suggesting that access to employee benefits, regardless of whether they were pro-family or not, is associated with reduced depressive symptoms among women in competing roles; and (4) there was no evidence that the effects were attributable to selection out of employment or into jobs with pro-family benefits.

Popularized as a result of the “second shift” faced by women,^1^ the mental health effects of holding competing roles are still an active area of research in the current social context. Holding multiple roles can be good for mental health and well-being^14^^‑^^16^^,^^64^; however, the present study found that competing roles were associated with increased depressive symptoms, suggesting that competition for the resources needed to satisfy both workplace and domestic labor roles may increase risk of depression.^11^ These findings are concordant with previous studies that have shown a detrimental effect of excessive role demands.^19^^‑^^22^^,^^64^^,^^65^ This competition has likely been increasing over time, as trends show that Americans work longer hours overall than they have in the past and earn a wage premium for overwork (more than 50 hours of work per week)^66^ and that women spend more time providing care for their children,^67^ even into adulthood.^68^ Most recently, the effects of the COVID-19 pandemic have accelerated the prevalence of competing demands^69^ and accentuated their mental health consequences for women.^70^

The buffering effects of pro-family employee benefits identified in this study are generally consistent with previous research and extend the evidence base to documenting benefits for depression symptoms. Access to paid family leave increases the use of preventative health care^71^ and decreases fatigue, anxiety, and depressive symptoms among new mothers.^72^ The use of flexible working policies improves mother-child bonding during early childhood,^73^ decreasing the mother’s depression risk in turn.^74^ Research on the impact of the availability of employer-provided or subsidized child care on employee mental health is very limited, though there is indirect evidence of positive mental health effects of this specific benefit. Increasing the affordability of childcare increases employee retention, which likely reduces the depression risk associated with job turnover.^75^^,^^76^

The presence of non–family-related employee benefits also was associated with reduced depression risk from competing roles, suggesting that the attenuation of stress from competing roles is not limited to benefits designed to specifically address role competition among working women with children. There are several potential explanations for these general positive effects. First, the availability of personal health insurance improves access to preventive care and treatment for those at risk of depression, including new mothers.^77^ Second, access to pensions and other retirement benefits decreases job changes and associated stress^43^ and may provide financial security among parents, who may be particularly concerned about future financial burdens. Third, jobs with a wide array of employee benefits are generally higher-quality jobs^28^^,^^78^; they signal greater occupational prestige^42^ and are associated with higher employee self-esteem and general satisfaction^41^ than jobs without benefits. All of these characteristics could potentially reduce stress and subsequent depression risk related to competing roles. Though we attempted to account for alternative explanations by adjusting model estimates for work hours, income, industry, and employer type, the nonspecific nature of these effects highlights a broader positive impact of workplace policies to reduce depressive symptoms among women with children. While descriptive in nature, the sensitivity analysis supported the explanation that availability of health insurance is broadly beneficial, as are family leave and flexible work scheduling benefits.

 The analytical sample was restricted to full-time employed respondents, which would cause selection bias if employment status were related to competing roles and depression. While we could not avoid this selection, given the study design, we attempted to account for the potential magnitude of the bias in a sensitivity analysis. To the extent that these selection pressures are measured and captured in the short term (ie, 2-year lags), the magnitude of selection bias in this study appeared to be minimal. Future research could further probe this relationship, particularly investigating differences among the full-time and part-time workforce. The prevalence of part-time, contingent, and precarious employment has increased in recent years, especially among women.^79^ Individuals in these jobs experience unique stressors at the intersection of the formal and informal labor forces, which may subsequently influence their mental health. The buffering effects of employee benefits may be especially beneficial for these workers. It is also possible that healthier individuals may be more likely to select into jobs with more benefits, though related evidence to date suggests that selection effects are secondary and are not likely to explain the results of this study.^80^ Indeed, the sensitivity analysis of selection into jobs with pro-family benefits found minimal evidence of bias. Increasing access to high-quality full- and part-time employment conditions may improve health equity among working adults and their families,^81^ and accounting for both selection differences and heterogeneity in these estimates across marginalized group identities^82^^,^^83^ is a critical step in closing socioeconomic disparities and their health consequences.

This research should be interpreted in light of several limitations. First, employee benefits were measured on the basis of self-report. There is evidence that employees’ knowledge of their workplace benefits is underestimated,^84^ which would introduce measurement error in these variables. It is plausible that employees with children (ie, those with competing roles) would report the availability of family leave policies more accurately than those without children.^84^ If recall was independent of depressive symptoms, interaction estimates may also have been attenuated. Regardless, the buffering effects estimated in this study may be best defined as the effect of the awareness of employee benefits, and in future research investigators should utilize a more objective measure of benefits (eg, human resources data) to clarify this potential measurement issue. This approach could be extended to include self-employed individuals. Second, while the National Longitudinal Survey does ask more detailed questions about childcare arrangements, it does not directly measure the amount of time spent providing childcare among respondents. However, since women still provide the majority of childcare on average,^7^^‑^^9^ the presence of a child in the home would likely be a valid proxy for increased domestic labor responsibilities. To further probe the degree of support available to mothers in the study sample, we calculated the number of additional sources of care (ie, when the child was not in the respondent’s care), including the respondent’s spouse, other relatives, unrelated adults, school/day-care personnel, after-school care personnel, or sibling(s). On average, women in competing roles reported 1.54 and 1.85 additional sources of care than women without (vs with) access to pro-family benefits, respectively (see Table 1), suggesting that the childcare involved multiple sources. Indeed, time spent on childcare has been increasing among male partners.^67^^,^^85^ The mental health effects among women (and men) who experience more equitable or male-dominated domestic labor arrangements may be positive^86^ or negative,^87^ but more research is needed in the face of changing childcare trends. Third, in addition to childcare needs, caregiving for aging family members has also grown in recent years.^88^ Historically, women have provided most of this care,^89^ although men are increasingly involved.^90^ Inclusion of all caregiving relationships may provide a more accurate estimate of competing roles; however, the effects of caring for an adult may differ from those of childcare. Future work could elucidate the common and distinct health risks across different types of caregiving roles. Finally, depression scores as measured in this study refer to short-term (2-week) prevalence, which may not reflect true depression status over the ≥2-year period between interviews or capture depression incidence. While the longitudinal design did establish temporality at each interview, a study with a younger age of follow-up and ascertainment of incident depression cases would help to further our understanding of the risk of competing roles.

Despite these limitations, this study was strengthened by the inclusion of a large cohort of women covering a wide age range that probably captured the ages at which employment and domestic roles were most likely to be in competition. Additionally, inclusion of a wide array of employee benefits allowed for examination of cumulative buffering effects of multiple benefits on competing roles and MHI-5 scores, and a comparison of the effects with those of non–family-related benefits.

In conclusion, patterns of women’s participation in the workplace and domestic labor suggest that competition from dual workplace and domestic labor roles may represent a risk factor for depression for women. Working women with children inevitably face overlap in the responsibilities that both roles demand, and although workplace benefits may help to attenuate that risk, more fundamental social changes are needed to address the residual sex/gender inequalities in paid and unpaid labor roles in the United States.

## Supplementary Material

Web_Material_kwae055

## Data Availability

National Longitudinal Survey data are managed by the US Bureau of Labor Statistics and are publicly available at the following website: https://www.nlsinfo.org/investigator/pages/login.
