# Supplementary material for: Bringing home the benefits: do pro-family employee benefits mitigate the risk of depression from competing workplace and domestic labor roles?
Source: Am J Epidemiol. 2024 Apr 26;193(10):1362–71. doi: 10.1093/aje/kwae055 (PMC11458195; doi:10.1093/aje/kwae055)
Supplement: Web_Material_kwae055 [file web_material_kwae055.pdf]

Supplementary Material

Bringing home the benefits: Do pro-family employee benefits mitigate the risk of depression from competing workplace and domestic labor roles?

Jonathan M. Platt, Lisa Bates, Justin Jager, Katie A. McLaughlin, Katherine M. Keyes

Contents

Figure S1 2
Table S1 3
Table S2 4
Table S3 5
Table S4 6

Figure S1. Study flow chart describing sample sizes at each interview.

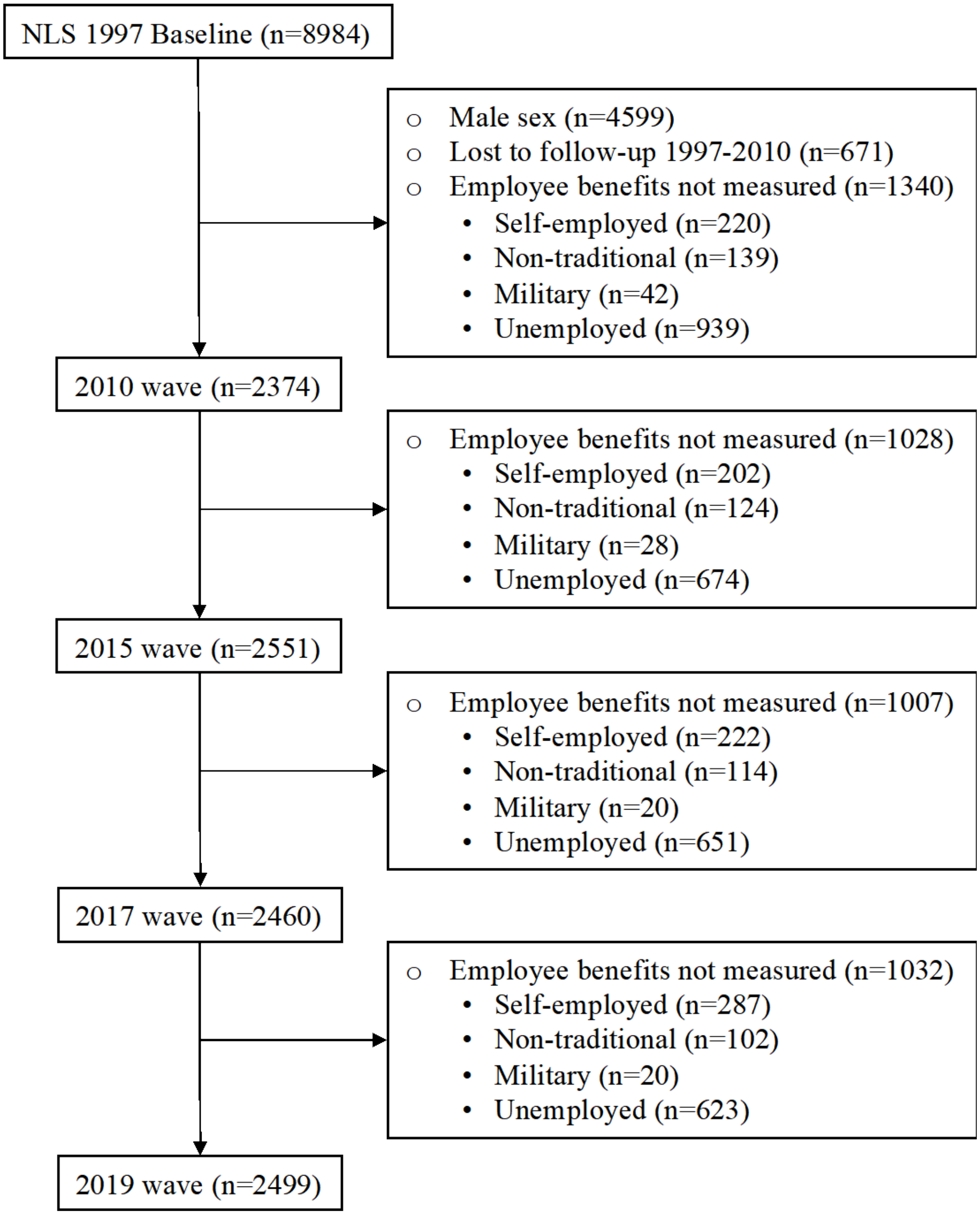

Note: *non-traditional* status includes temporary, contract-based, or other on-call employment.

Table S1. Distribution of industries in the total analytical sample

Industry	No.	%
1. Agriculture, Forestry, Fishing, and Hunting	48	0.4
2. Mining	17	0.14
3. Utilities	67	0.56
4. Construction	172	1.44
5. Manufacturing	1044	8.74
6. Wholesale Trade	276	2.31
7. Retail Trade	932	7.8
8. Transportation and Warehousing	423	3.54
9. Information	267	2.23
10. Finance and Insurance	783	6.55
11. Real Estate and Rental and Leasing	186	1.56
12. Professional, Scientific, and Technical Services	500	4.18
13. Management, Administrative and Support, and Waste Management Services	409	3.42
14. Educational Services	1519	12.71
15. Health Care and Social Assistance	2774	23.22
16. Arts, Entertainment, and Recreation	126	1.05
17. Accommodations and Food Services	492	4.12
18. Other Services (Except Public Administration)	366	3.06
19. Public Administration and Active Duty Military	1013	8.48
20. Armed Forces (for CPS)	12	0.1
21. No code	813	6.64

Table S2. MHI-5 symptom score differences among women in competing roles vs. not in competing roles, stratified by the availability (any vs. none) and a count of pro-family employee benefits, 2010-2019

Availability of Pro-Family Benefits	MHI-5 Score Difference (95% CI)*[a]
No benefits	6.1 (1.14, 11.1)
Any benefits (ref=none)	0.44 (-0.21, 1.0)
One benefit (ref=none)	1.1 (-0.08, 2.26)
Two or more benefits (ref=none)	-0.01 (-0.87, 0.86)

Note: Competing roles are defined as working with children living in the respondent's household (ref=working with no children living in the household); MHI=Mental Health Inventory scale; CI=Confidence Interval. Interaction test: H0: competing roles x benefits=0.

*Adjusted for age, race/ethnicity, hours of paid work per week, employer type, industry, education, number of children, children under 5, and non-family-related benefits.

[a] Interaction B=-0.51, p=0.017 without adjustment for non-family-related benefits; B=-0.44, p=0.023 with adjustment for non-family-related benefits.

Table S3. MHI-5 symptom score differences among women in competing roles vs. not in competing roles, stratified by the availability of any (any vs. none) and a count of non-family-related employee benefits, 2010-2019

Availability of Non-Family-Related Benefits	MHI-5 Score Difference (95% CI)*
No benefits	3.59 (1.24, 5.95)
Any benefits (ref=none)	0.57 (-0.61, 1.74)
One benefit (ref=none)	2.09 (-0.26, 4.44)
Two or more benefits (ref=none)	0.44 (-0.73, 1.62)

Note: Competing roles are defined as working with children living in the respondent's household (ref=working with no children living in the household); MHI=Mental Health Inventory scale; CI=Confidence Interval.

*Adjusted for age, race/ethnicity, hours of paid work per week, employer type, industry, education, number of children, children under 5, and pro-family benefits.

Table S4. Interaction coefficients between competing roles and individual employee benefits

Benefit	Parameter (99% CI)
Dental	-0.32 (-0.5, -0.15)
Life insurance	-0.26 (-0.43, -0.08)
Medical insurance	-0.41 (-0.6, -0.22)
Profit sharing	-0.08 (-0.28, 0.13)
Retirement	-0.16 (-0.34, 0.02)
Training/education	-0.29 (-0.45, -0.13)
Childcare	-0.23 (-0.52, 0.06)
Family leave	-0.19 (-0.37, -0.01)
Flexible work scheduling	-0.18 (-0.34, -0.02)
